# From Sir Henry Burdett, K.C.B.

**Published:** 1918-07-27

**Authors:** Henry C. Burdett

**Affiliations:** The Lodge, 13 Porchester Square, W. 2


### From Sir Henry Burdett, K.C.B.

SIR,—Lord Knutsford's letter in *The Times* to-day does not touch my points, and is mere camouflage. I became a life governor of the London Hospital some years before Lord Knutsford was associated with it, and I yield to no one in upholding its honour and promoting its advancement. The admittedly good training of its nurses is not in question, but where do the rights of the probationers come in? The fact that the Matrons-in-Chief in England and in France are both London Hospital trained implies the holding of a three years' certificate of training. The London Hospital has issued, and does issue, a three years' certificate of training, the whole of which period has been spent in practical work in the London Hospital, or it would have been impossible for Lord Knutsford to quote the figures given in his letter of the 15th instant. On what grounds, therefore, does the Chairman of the London Hospital refrain from announcing that, in future, probationers may have the option of entering for three years' training, to be spent in practical work in that institution? Such a step would be calculated to lessen any shortage of probationers and add to the deservedly high reputation of the London Hospital. It would demonstrate, too, the statesmanship of Lord Knutsford himself. I may add that neither Colonel Maurice nor myself had any knowledge of each other's letters until we saw them in the columns of *The Times*.—Yours faithfully,

HENRY C. BURDETT.

The Lodge, 13 Porchester Square, W. 2, July 16.

